# Synthesis of ultrathin platinum nanoplates for enhanced oxygen reduction activity†
†Electronic supplementary information (ESI) available: NMR spectra, TEM images, HAADF-STEM images, ED patterns, XRD patterns, UV-vis spectra, electrocatalytic results, and additional discussion on these results. See DOI: 10.1039/c7sc02997g


**DOI:** 10.1039/c7sc02997g

**Published:** 2017-10-30

**Authors:** Hongpo Liu, Ping Zhong, Kai Liu, Lu Han, Haoquan Zheng, Yadong Yin, Chuanbo Gao

**Affiliations:** a Center for Materials Chemistry , Frontier Institute of Science and Technology and State Key Laboratory of Multiphase Flow in Power Engineering , Xi’an Jiaotong University , Xi’an , Shaanxi 710054 , China . Email: gaochuanbo@mail.xjtu.edu.cn; b School of Chemistry and Chemical Engineering , Shanghai Jiao Tong University , Shanghai 200240 , China; c School of Chemistry and Chemical Engineering , Shaanxi Normal University , Xi’an , Shaanxi 710119 , China; d Department of Chemistry , University of California , Riverside , California 92521 , USA . Email: yadong.yin@ucr.edu

## Abstract

Ultrathin Pt nanostructures exposing controlled crystal facets are highly desirable for their superior activity and cost-effectiveness in the electrocatalytic oxygen reduction reaction (ORR), and they are conventionally synthesized by epitaxial growth of Pt on a limited range of templates, such as Pd nanocrystals, resulting in a high cost and less structural diversity of the ultrathin Pt nanostructures. To solve this problem, we demonstrate that ultrathin Pt nanostructures can be synthesized by templating conveniently available Ag nanocrystals without involving galvanic replacement, which enables a much-reduced cost and controllable new morphologies, such as ultrathin Pt nanoplates that expose the {111} facets. The resulting ultrathin Pt nanoplates are ∼1–2 nm in thickness, which show an ∼22-fold increase in specific activity (5.3 mA cm^–2^), an ∼9.5-fold increase in mass activity (1.62 A mg^–1^) and significantly enhanced catalytic stability in the ORR, compared with the commercial Pt/C catalyst. We believe this strategy opens a door to a highly extendable family of ultrathin noble metal nanostructures, thus promising excellent activity and stability in a broad range of catalytic applications.

## Abstract 2

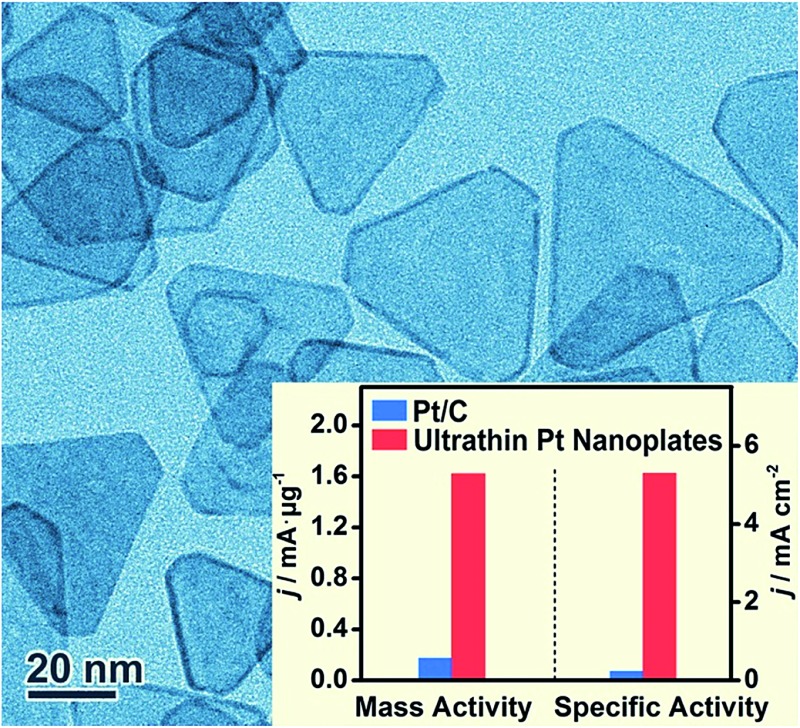
Ultrathin Pt nanoplates exposing {111} facets are synthesized by galvanic-replacement-free templating of conveniently available Ag nanoplates, showing enhanced oxygen reduction activity.

## Introduction

Ultrathin Pt and Pt-alloy nanostructures have attracted increasing interest in recent years due to the extremely high fraction of metal atoms at the surface and thus efficient utilization of metal active sites in pursuit of high catalytic activity at a low cost, especially in the oxygen reduction reaction (ORR), the rate-limiting step in a proton-exchange membrane fuel cell.^1^–^18^ The synthesis of thin noble metal nanostructures was pioneered in the last decade by wet chemical synthesis of nanoplates.^19^–^28^ As the formation of these nanoplates relies on the delicate modulation of the capping effect of ligands on specific facets of noble metal nanocrystals, the success has been largely limited to a few metals so far, such as Au,^19^,^20^ Ag,^21^–^23^ Pd,^24^ Rh^25^–^27^ and Ru,^28^ and the thickness is not readily tunable on demand for optimizing the catalytic activity. To address this problem, a hard-templating strategy has been developed, with ultrathin noble metal nanostructures obtained by epitaxial growth of metals on a sacrificial template.^1^–^9^,^29^–^31^ The introduction of the templates extends the ultrathin nanostructures to a much broader range of metals, including Pt, and enables flexibly tweakable exposed facets and thickness from angstroms to a few nanometers. However, state-of-the-art templates for the synthesis are limited to graphene^29^–^31^ and precious Pd nanocrystals,^1^–^9^ which afford ultrathin Pt nanostructures with limited structural diversity and a high cost. So far, there remains a great margin in the synthesis of ultrathin Pt nanostructures with alternative templates that are much more economical and diverse in morphology and facet structure.

Typically, Ag nanocrystals represent an appropriate choice for the templates due to the high abundance of Ag in the earth’s crust (∼70 ppb) and hence relatively low price, the well tunable morphology, and the robust scalable production.^21^,^22^,^32^–^38^ However, Ag nanocrystals as templates are usually susceptible to galvanic replacement with a chloroplatinate salt, leading to hollow nanostructures:^39^–^42^PtCl_4_^2–^ + 2Ag ⇋ Pt + 2AgCl + 2Cl^–^, *E*_0_ = 0.536 V (*vs.* SHE)PtCl_6_^2–^ + 4Ag ⇋ Pt + 4AgCl + 2Cl^–^, *E*_0_ = 0.520 V (*vs.* SHE)

Although strategies have been developed to suppress this galvanic replacement, including thermodynamic control *via* tuning the reduction potentials of the noble metal salts by coordinating to a ligand^43^–^45^ and kinetic control *via* balancing the competing rates of the crystal growth against the galvanic replacement,^46^–^51^ the success has been largely restricted to the synthesis of Ag@Au core/shell nanocrystals. To date, only very limited progress has been made on the epitaxial growth of Pt on sacrificial Ag nanocrystals to synthesize ultrathin Pt nanostructures for distinctive catalytic properties.^50^–^52^

Herein, we demonstrate a galvanic-replacement-free epitaxial growth of Pt on Ag nanocrystals, nanoplates for example, by combining control over the reaction thermodynamics and kinetics, and therefore the successful synthesis of a novel family of ultrathin truncated triangular Pt nanoplates enclosed by {111} facets after etching of Ag. Although ultrathin Pt nanostructures can be obtained by the templating of Pd nanocrystals, ultrathin Pt nanoplates have been synthesized for the first time enabled by galvanic-replacement-free templating of conveniently available Ag nanoplates. Thanks to the ultrasmall thickness,^1^–^18^ the exposure of a favorable {111} facet^53^,^54^ and structural defects,^4^–^6^,^11^,^16^,^55^ the presence of a low fraction Ag^40^ and the self-supported structure,^39^ these ultrathin Pt nanoplates exhibit significantly enhanced activity and stability in the ORR, benchmarking against the commercial Pt/C catalyst. Therefore, this unique strategy allows a robust, cost-effective synthesis of a new Pt nanocatalyst with distinctive morphology and an exceptional {111} exposing facet for enhanced oxygen reduction activity.

## Results and discussion

The key to the suppression of the galvanic replacement between the Ag nanoplates and the chloroplatinate salt was two-fold. First, the reduction potential of the chloroplatinate salt was significantly decreased by coordinating to CH_3_CN to thermodynamically suppress the tendency for galvanic replacement (Fig. S1–S4, ESI†). Second, a strong reductive environment was applied by introducing a combination of deprotonated ascorbic acid and high-temperature H_2_,^47^–^49^,^56^ which leads to a favorable reduction of the H_2_PtCl_6_/CH_3_CN complex and thus competitive kinetics of the crystal growth against the potential galvanic replacement (Fig. S5 and S6, ESI†). In addition, polyvinylpyrrolidone (PVP) has been introduced as a capping agent to further enhance the stability of the Ag nanoplates (Fig. S7, ESI†).^43^ By taking advantage of this delicately designed seeded growth system, continuous epitaxial growth of Pt on the surface of Ag nanoplates has been successfully achieved without involving galvanic replacement.

**Fig. 1:**
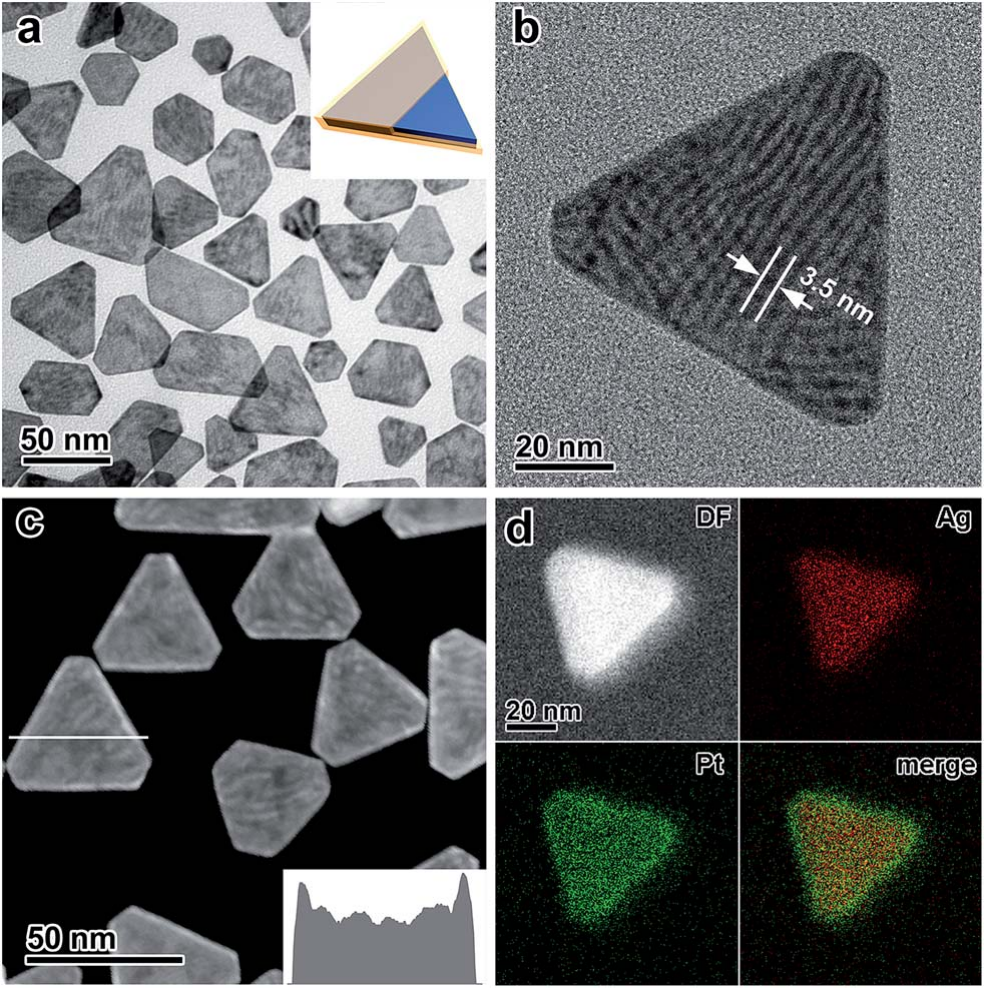
(a) Low-magnification TEM image of the Ag@Pt core/shell nanoplates. Inset: a model of the nanostructure. (b) Moiré fringes observed from a single Ag@Pt core/shell nanoplate. (c) HAADF-STEM image of the Ag@Pt core/shell nanoplates. Inset: profile of the intensity across a nanoplate along the white line as indicated in the image. (d) EDS elemental mapping of a single Ag@Pt core/shell nanoplate.

The absence of the galvanic replacement between the Ag nanoplates and the Pt salt can be confirmed by transmission electron microscopy (TEM) imaging, which shows the absence of any voids at the surface of the Ag nanoplates (Fig. 1a). On each Ag@Pt core/shell nanoplate, Moiré fringes are observable as parallel strip patterns with an average spacing of ∼3.5 nm (Fig. 1b), which can be ascribed to the mismatch of the {220} crystal planes between the Ag and Pt layers, in good agreement with the theoretical value (3.47 nm). The core/shell contrast can be clearly observed from the high-angle annular dark field scanning transmission electron microscopy (HAADF-STEM) image (Fig. 1c and S8, ESI†). It shows a dark triangular zone in the center enclosed by a bright zone at the edge of an individual nanoplate, which can be ascribed to the Ag core and the Pt shell, respectively. The difference in the image brightness can be described quantitatively by an intensity profile across a single nanoplate, confirming the enrichment of Pt at the edges of the nanoplates (Fig. 1c, inset). The energy-dispersive X-ray spectroscopy (EDS) provides additional information about the core/shell nanostructure in an elemental mapping analysis, with the Ag core and the Pt shell unambiguously distinguished with a clear Pt/Ag interface in a single nanoplate (Fig. 1d). The thickness of the Pt shell at the edge of the Ag nanoplates can be estimated to be ∼2 nm from both the HAADF-STEM and the EDS elemental mapping images.

**Fig. 2:**
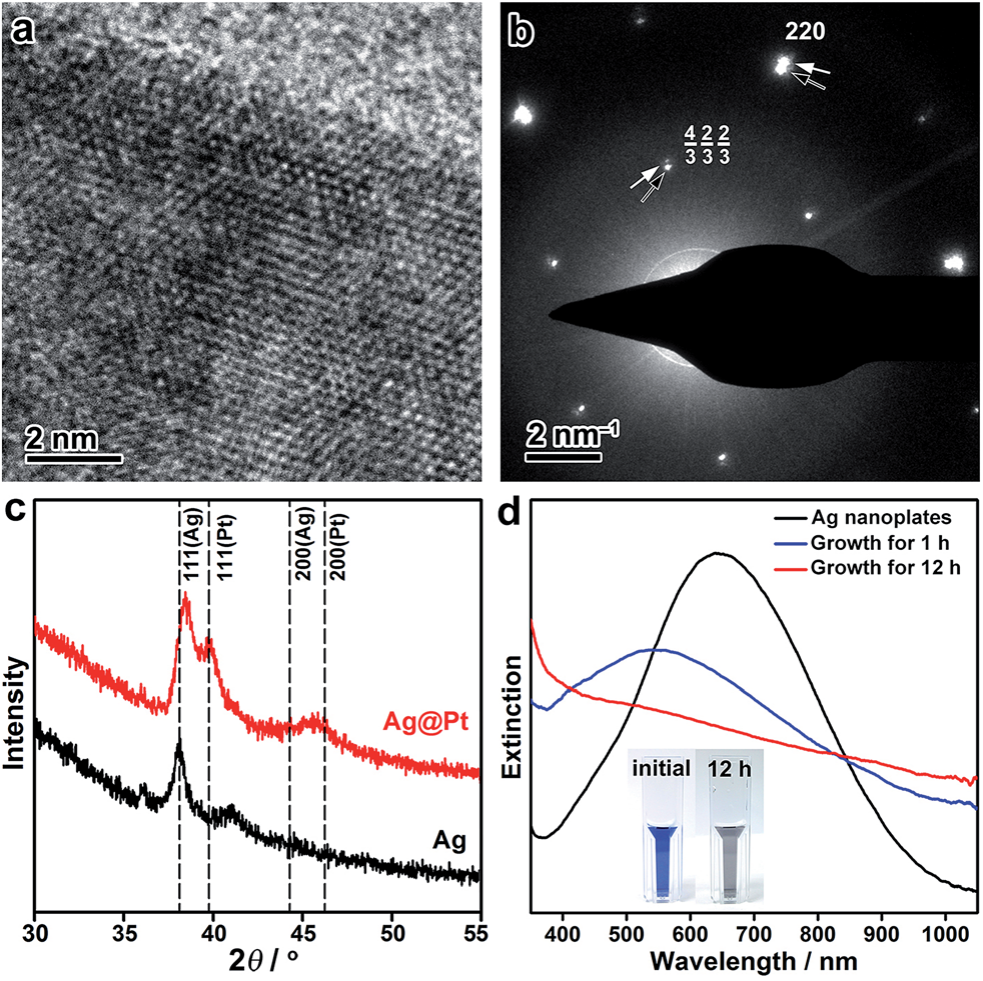
(a) HRTEM image of a Ag@Pt core/shell nanoplate at its edge. (b) ED pattern taken from a single nanoplate. Black and white arrows indicate the diffractions from Ag and Pt, respectively. (c) XRD patterns of the Ag nanoplates and the Ag@Pt core/shell nanoplates. Standard positions for Ag and Pt are indicated by dashed lines. (d) Evolution of the UV-vis spectroscopy during the epitaxial growth of Pt on the Ag nanoplates. Inset: digital photographs of the Ag nanoplates before and after the growth of Pt.

The crystal structure of the Ag@Pt core/shell nanoplates was further inspected by high-resolution TEM (HRTEM), electron diffraction (ED), and X-ray diffraction (XRD) techniques. The HRTEM image shows distinctive single crystallinity, without an observable clear difference in the crystal orientation at the Ag/Pt interface (∼2 nm from the edge), confirming the epitaxial growth of Pt on the Ag lattice (Fig. 2a). The ED pattern taken from a single Ag@Pt core/shell nanoplate clearly demonstrates two sets of ED spots, which can be ascribed to the diffractions from the Ag and Pt layers, respectively (Fig. 2b and S9, ESI†). The two sets of diffractions are accompanying each other at the same orientation relative to the primary beam, confirming the epitaxial crystal growth. It is interesting that the formally forbidden 1/3{422} diffractions of the Pt layer are well discernible from the ED pattern, which is indicative of planar defects formed during the epitaxial crystal growth.^57^,^58^ Consistently, the XRD pattern of the core/shell nanoplates shows two sets of X-ray reflections that correspond to Ag and Pt, respectively (Fig. 2c). A slight deviation in the position of the (111) reflection from Ag can be noticed, which might arise from partial interfacial alloying of Ag with Pt during the epitaxial growth. Both the ED and the XRD patterns suggest that the Pt layer retains its intrinsic lattice parameters after extensive epitaxial growth on the Ag nanoplates.

The seeded growth of Pt on the Ag nanoplates can be monitored by UV-vis spectroscopy (Fig. 2d). Pristine Ag nanoplates showed an initial in-plane dipole-mode localized surface plasmon resonance (LSPR) band at ∼640 nm of the wavelength. At the early stage of the epitaxial growth of Pt on the Ag nanoplates (after 1 h of growth), a blue-shift of the LSPR can be witnessed with a decreased extinction efficiency. It is worth noting that galvanic replacement usually causes a red-shift of the LSPR band due to the hollowing of the nanostructures. This distinct shift of the LSPR band confirmed the absence of galvanic replacement in the epitaxial growth. After deposition of an appropriately thick layer of Pt (∼2 nm, after 12 h of growth), the LSPR band disappeared in the visible range of the spectrum. As a result, the resulting Ag@Pt core/shell nanoplates displayed a grey/black color. Therefore, the optical property of the metal nanoplates can be utilized as an indicator to inspect the crystal growth process.

**Fig. 3:**
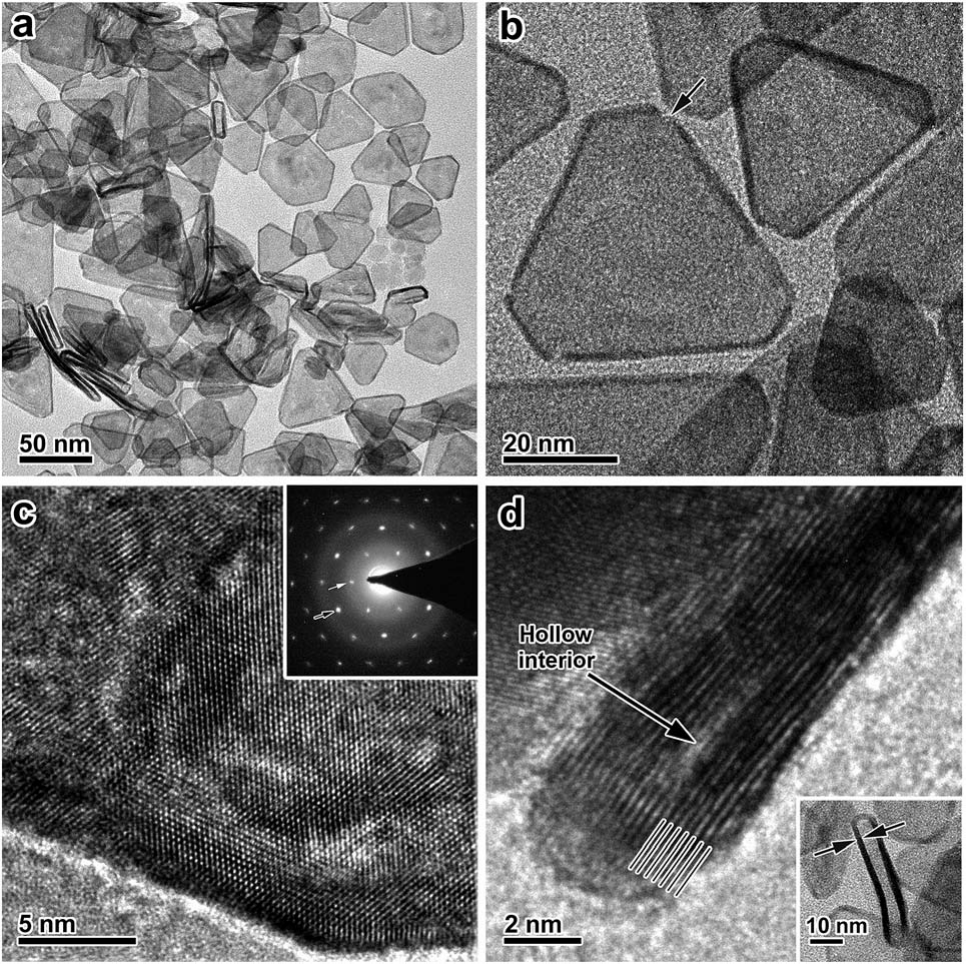
Characterizations of the ultrathin Pt nanoplates. (a) Low-magnification TEM image of the ultrathin Pt nanoplates. (b) TEM image of the ultrathin Pt nanoplates. The arrow indicates a defect. (c) HRTEM image of an individual ultrathin Pt nanoplate imaged with an electron beam perpendicular to the basal {111} facet. Inset: the typical ED pattern. Black and white arrows indicate the {220} and the formally forbidden 1/3{422} diffractions, respectively. (d) HRTEM image of a vertically aligned ultrathin Pt nanoplate. Lines indicate the alignment of the atoms along the [111] direction. Inset: a low-magnification TEM image.

After the galvanic-replacement-free epitaxial growth of Pt on the Ag nanoplates, the Ag templates are etched by an oxidizing agent to eventually afford the ultrathin Pt nanoplates. During the initial stage of the epitaxial growth, intermixing of Pt and Ag occurs, which gives rise to pinholes in the epitaxial layer of Pt.^3^ These pinholes enable the selective etching of the Ag templates by an oxidizing agent, nitric acid (HNO_3_) for example, and therefore ultrathin Pt nanoplates can be obtained in a high yield (Fig. 3a and b). These ultrathin Pt nanoplates are mainly composed of Pt with a molar fraction of ∼91%, as determined by inductively coupled plasma mass spectrometry (ICP-MS). Ag was inevitably found in the Pt nanoplates because of the intermixing of Ag and Pt during crystal growth. The uniform alloying of Ag in the Pt nanoplates can be further confirmed by EDS elemental mapping (Fig. S11, ESI†). However, the fraction of Ag is minor (∼9%, significantly lower than those of ultrathin Pt nanostructures obtained by galvanic replacement), which can be attributed to the absence of galvanic replacement in this unique epitaxial growth mechanism. The low Ag content endows the ultrathin Pt nanoplates with a high stability in the acidic electrolyte when catalyzing the ORR reaction. These ultrathin Pt nanoplates show a hollow interior with pinholes clearly observable in the ultrathin Pt layers (Fig. 3b). The smooth surface of the nanoplates suggests a thermodynamic Frank–van der Merwe mode (layer-by-layer growth) crystal growth of Pt on the Ag surface, rather than a Volmer–Weber mode (island growth),^59^,^60^ albeit the large lattice mismatch (∼4.2%).^59^ This observation can be largely ascribed to the introduction of the ligand to the synthesis system, which adsorbs on both the Ag surface and the newly formed Pt surface, and thus the surface reactivity has been averaged. On the other hand, the ligand slows down the reduction rate of the Pt salt, which allows migration of the Pt atoms to distant regions during the growth, favorable for the formation of a smooth surface. As a result, Pt nanoplates with ultrathin thickness can be conveniently achieved. Due to the epitaxy in the crystal growth, the surface structure of the ultrathin Pt nanoplates can be determined by the Ag templates. The HRTEM images and ED patterns confirm that the ultrathin Pt nanoplates are single crystals exposing exclusive {111} facets (Fig. 3c and S12, ESI†). Two types of structural defect are possibly present in these ultrathin Pt nanoplates. First, the Ag in the Pt layer could be leached to an extent when exposed to the nitric acid, which may lead to a defective Pt{111} surface,^61^ as indicated by the inhomogeneous image contrast of the ultrathin Pt nanoplates (Fig. 3c). Second, stacking faults or twin defects are present in the [111] direction of the ultrathin Pt nanoplates, and are exposed at their side facets, as suggested by the presence of the formally forbidden 1/3{422} diffractions in the corresponding ED pattern (Fig. 3c, inset).^57^,^58^ The thickness of the double Pt layer in the ultrathin Pt nanoplates can be estimated to be ∼2 nm, which is approximately 7–9 layers of atoms along the [111] direction, according to the HRTEM image (Fig. 3d). It is worth noting that ultrathin Pt nanoplates of varying sizes can be achieved by following a similar process with Ag nanoplates of a specific size as the template (Fig. S13, ESI†).

**Fig. 4:**
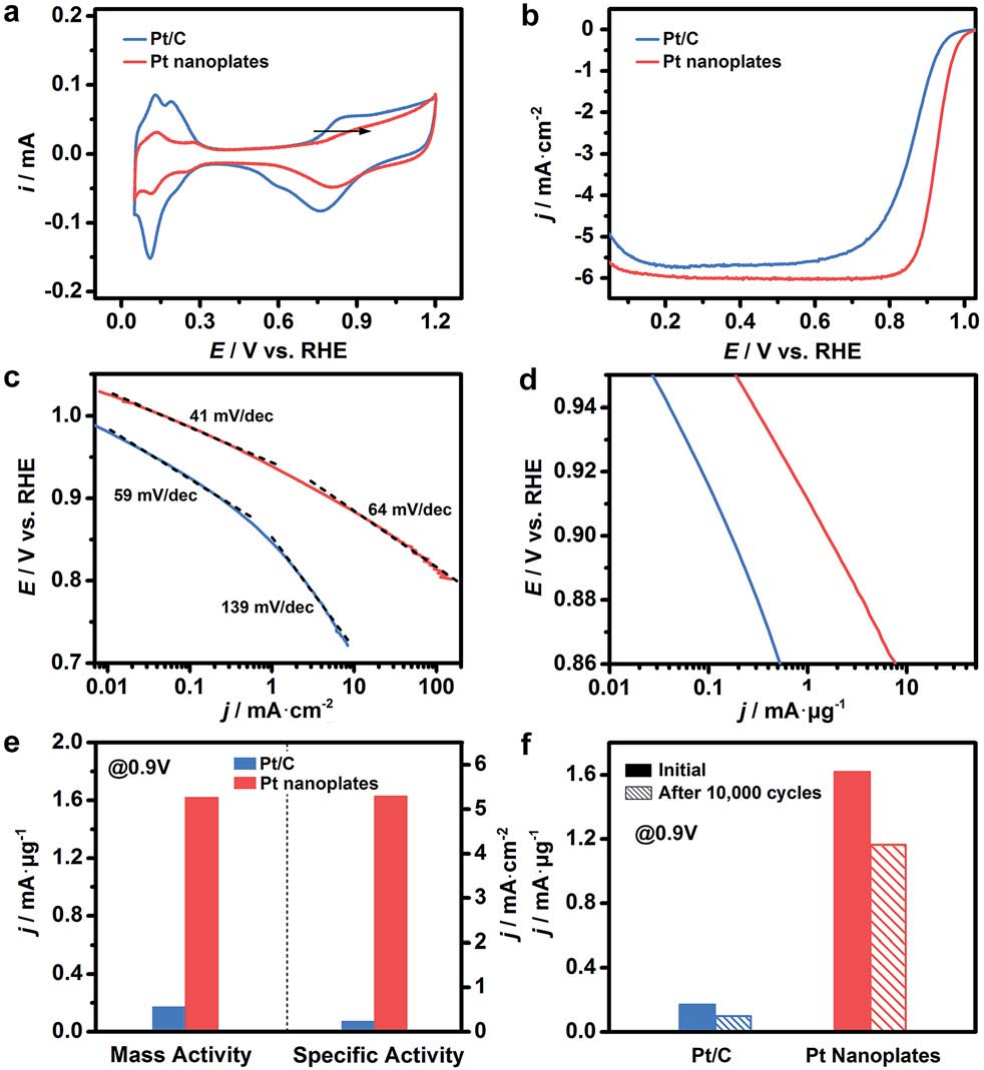
Electrocatalytic activity of the ultrathin Pt nanoplates in the ORR, benchmarked against the commercial Pt/C catalyst. (a) CV curves of the catalysts in N_2_-saturated HClO_4_ (0.1 M) at a sweep rate of 50 mV s^–1^. The arrow indicates the positive shift of the OH* adsorption peak on the Pt nanoplates relative to that of the Pt/C. (b) ORR polarization curves of the catalysts in O_2_-saturated HClO_4_ (0.1 M) at a sweep rate of 10 mV s^–1^ and rotation speed of 1600 rpm. The current densities were normalized to the geometric area of the rotating disk electrode (0.196 cm^2^). (c and d) The corresponding Tafel plots. The specific and mass activities of the catalysts given as kinetic current density (*j*_k_) were normalized to the ECSA and Pt mass, respectively. (e) Mass and specific activities of the ultrathin Pt nanoplates at 0.9 V *vs.* RHE, in comparison with the commercial Pt/C catalyst. (f) Comparison of mass activities of the catalysts at 0.9 V *vs.* RHE before and after the accelerated durability tests.

These ultrathin Pt nanoplates represent a novel family of electrocatalyst that promises high activity and durability in practical electrocatalytic applications. To demonstrate this, we evaluated the electrocatalytic property of the ultrathin Pt nanoplates in the ORR by benchmarking against the commercial Pt/C catalyst (JM, Pt 20%) (Fig. 4). The electrochemically active surface areas (ECSAs) of the ultrathin Pt nanoplates and the commercial Pt/C can be determined to be 30.6 and 71.9 m^2^ g^–1^, respectively (Fig. 4a). Fig. 4b shows the positive-going ORR polarization curves of the catalysts in O_2_-saturated HClO_4_ (0.1 M) at a sweep rate of 10 mV s^–1^ and a rotation rate of 1600 rpm after *iR* and background current corrections. It is inferred that the half-wave potential of the ultrathin Pt nanoplates (0.926 V) is much more positive than that of the commercial Pt/C (0.880 V), indicating superior catalytic activity of the ultrathin Pt nanoplates. For quantitative comparison, the kinetic current densities were calculated by the Koutecky–Levich equation normalized to the ECSA and Pt mass of the catalysts, respectively (Fig. 4c and d). The specific activity Tafel slopes of the ultrathin Pt nanoplates are ∼41 mV dec^–1^ in the high potential region (low current density) and ∼64 mV dec^–1^ in the low potential region (high current density), which are much lower than those observed from the commercial Pt/C catalyst, ∼59 and ∼139 mV dec^–1^, respectively, suggesting significantly improved kinetics for the ORR (Fig. 4c). Impressively, the specific activity of the ultrathin Pt nanoplates reached 5.3 mA cm^–2^ at 0.9 V, which is ∼22 times that of the commercial Pt/C (0.24 mA cm^–2^) (Fig. 4e). The mass activity of the ultrathin Pt nanoplate was 1.62 mA μg^–1^ at 0.9 V, which is ∼9.5 times that of the commercial Pt/C catalyst (0.17 mA μg^–1^) (Fig. 4d and e).

The difference in the Tafel slopes indicates that the ORR kinetics of the ultrathin Pt nanoplates are different from that of a commercial Pt/C catalyst. According to a density functional theory (DFT) calculation in a recent report,^62^ the Tafel slope observed from the ultrathin Pt nanoplates is consistent with the value obtained when the decomposition of the peroxy intermediate (OOH* + * → O* + OH*) is assigned as the rate-limiting step. This suggests that the adsorption energy of OH* on these ultrathin Pt nanoplates has been greatly weakened, and the hydrogenation of OH* is no longer a big barrier for the overall ORR reaction. The different adsorption energies of OH* on the Pt catalysts can be confirmed by the cyclic voltammograms (CV) (Fig. 4a).^61^ The OH* adsorption peak of the ultrathin Pt nanoplates at ∼0.8 V becomes weaker and shifts to a more positive potential relative to that of the Pt/C, which is a clear sign of the weakened adsorption energy of OH* on the ultrathin Pt nanoplates. We believe that the significantly weakened OH* adsorption energy is the primary reason for the high ORR kinetics on the ultrathin Pt nanoplates. Two structural features may account for the weakened adsorption energy of OH* on the ultrathin Pt nanoplates. First, the ultrathin Pt nanoplates selectively expose the {111} facet which binds much more weakly to OH* than many other facets such as {100}.^53^,^54^ Second, the defective Pt{111} surface and the twin defects on the side facets may cause a change in the coordination number of Pt^61^ and surface strain,^4^–^6^,^11^,^16^,^55^ respectively, which may further decrease the adsorption energy of OH* on the Pt surface. In addition, the presence of a low fraction of Ag in the ultrathin Pt nanostructures (∼9%) is favorable for the decomposition of the OOH* intermediate due to a synergistic ligand effect,^40^ leading to further enhancement of the ORR kinetics.

**Fig. 5:**
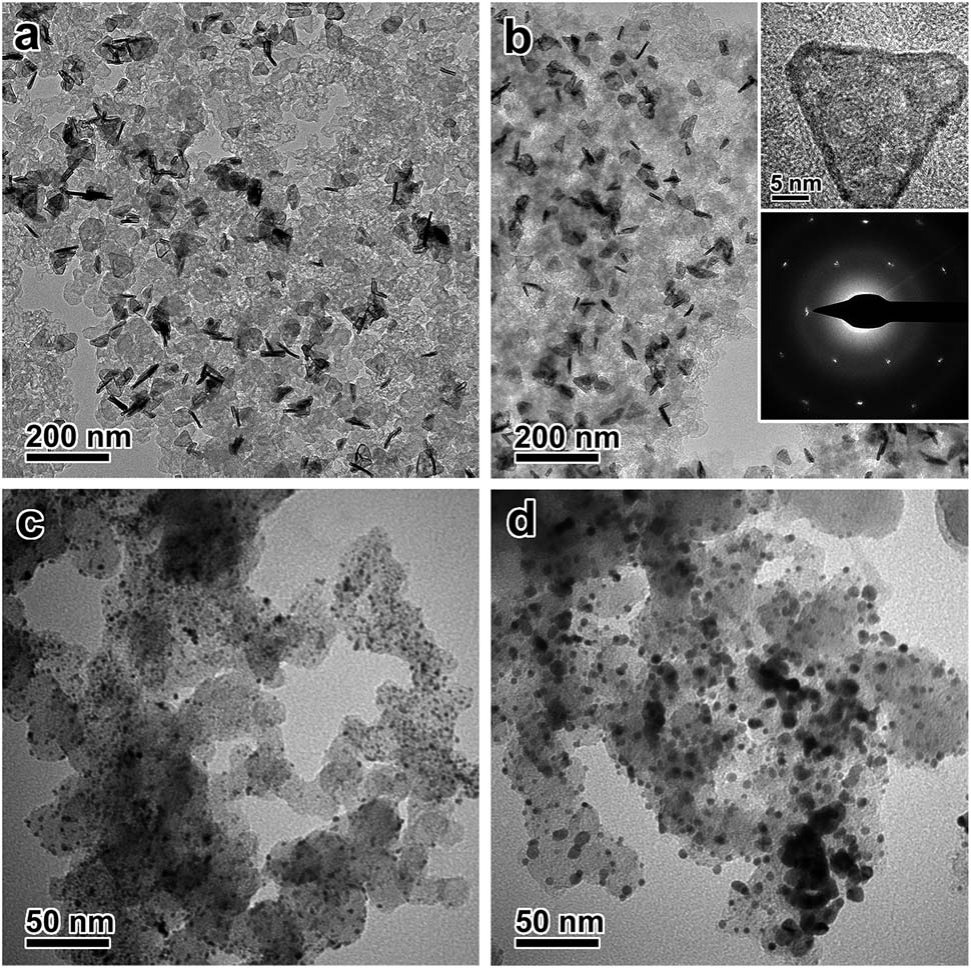
TEM images of the ultrathin Pt nanoplates and the commercial Pt/C catalyst before and after 10 000 cycles of potential sweeps in the ORR. (a) Pristine ultrathin Pt nanoplates supported on carbon black. (b) Ultrathin Pt nanoplates after the cycling process. Inset: HRTEM of a single nanoplate and the corresponding ED pattern. (c) Pristine Pt/C. (d) Pt/C after the cycling process.

The catalytic stability of the catalysts was evaluated by an accelerated test (Fig. 4f, S15 and S16, ESI†). After 10 000 cycles of potential sweeps, the ECSAs of the ultrathin Pt nanoplates dropped by ∼17.0%, whereas that of the commercial Pt/C decreased by ∼63.6%, confirming the high tendency of the self-supported ultrathin Pt nanostructures to retain their ECSAs during the cycling process (Fig. S15 and S16, ESI†). After 10 000 cycles, the ultrathin Pt nanoplates exhibit mass activities of 1.17 mA μg^–1^ in the ORR, which was ∼11.7 times that of the commercial Pt/C (0.10 mA μg^–1^) (Fig. 4f). TEM imaging reveals that the ultrathin Pt nanoplates show remarkable structural integrity and dispersity on the carbon support during the cycling process, which accounts for their superior catalytic stability in the accelerated durability test (Fig. 5a and b). For comparison, significant agglomeration of the Pt nanoparticles can be observed in the Pt/C catalyst during the cycling process (Fig. 5c and d), leading to a loss of the ECSA and thus the overall activity.

## Conclusions

In summary, we have demonstrated for the first time the successful epitaxial growth of Pt on Ag nanoplates without involving galvanic replacement by delicately tuning the reaction thermodynamics and kinetics in a seeded growth system, and consequently the production of ultrathin Pt nanoplates with novel morphology and {111} exposed facets at a low cost. These ultrathin Pt nanoplates exhibit excellent electrocatalytic activity and durability in the ORR, with the specific and mass activity being ∼22 and 9.5 times those of commercial Pt/C catalyst, respectively, which can be attributed to their ultrasmall thickness, the exposure of a favorable {111} crystal facet and abundant structural defects, the presence of a low fraction Ag and the self-supported nanostructure. This unique synthesis strategy extends the range of templates for the synthesis of ultrathin Pt nanostructures to a huge family of Ag nanocrystals, leading to much more diverse morphologies and facet structures of ultrathin Pt nanostructures as well as a much-reduced cost, compared with conventional methods with Pd nanocrystal templates. We believe that this strategy is highly extendable to the synthesis of a series of ultrathin noble metal nanostructures for optimal activity and stability in a broad range of catalytic reactions.

## Experimental

### Materials

Silver nitrate (AgNO_3_), chloroplatinic acid hexahydrate (H_2_PtCl_6_·6H_2_O), l-ascorbic acid (AA), acetonitrile (CH_3_CN), sodium hydroxide (NaOH), polyvinylpyrrolidone (PVP) (*M*_w_ 55 000), sodium borohydride (NaBH_4_), trisodium citrate dihydrate (TSC), hydrogen peroxide (H_2_O_2_, 30%), perchloric acid (HClO_4_, 70%), and nitric acid (HNO_3_, 70%) were purchased from Sigma-Aldrich. All chemicals were used as purchased without further purification.

### Synthesis of Ag nanoplates

Ag nanoplates (∼40 nm) were synthesized by a modified chemical reduction method.^21^,^22^ Typically, aqueous solutions of TSC (0.075 M, 12 mL), AgNO_3_ (0.1 M, 200 μL) and H_2_O_2_ (30%, 480 μL) were added into 200 mL of H_2_O in sequence. Then a freshly-made NaBH_4_ solution (0.1 M, 1.2 mL) was quickly injected into the above solution under vigorous stirring. After stirring for 30 min a sol of the Ag nanoplates was collected as a stock solution (Ag: ∼9.4 × 10^–5^ M).

### Synthesis of Ag@Pt core/shell nanoplates

In a typical synthesis of Ag@Pt core/shell nanoplates, 540 mL of the Ag nanoplates (∼40 nm, Ag: ∼9.4 × 10^–5^ M) were centrifuged and redispersed in 30 mL of H_2_O. Then 13 mL of CH_3_CN, 4 mL of PVP (5 wt%), 800 μL of AA (0.5 M), 800 μL of NaOH (1 M) and 200 μL of H_2_PtCl_6_ (0.1 M) were added to this solution in sequence. The reaction system was transferred to a high-pressure tube, filled with H_2_ at atmospheric pressure, and stirred at 120 °C for 12 h. Finally, Ag@Pt core/shell nanoplates were collected by centrifugation, washed twice with H_2_O, and redispersed in 10 mL of H_2_O.

### Synthesis of ultrathin Pt nanoplates

Ultrathin Pt nanoplates were obtained by etching the Ag templates from the Ag@Pt core/shell nanoplates. In a standard procedure, 10 mL of concentrated HNO_3_ was added to 10 mL of the sol of the Ag@Pt core/shell nanoplates, which was kept undisturbed at room temperature for 30 min. The product of ultrathin Pt nanoplates was collected by centrifugation and washed 4 times with H_2_O.

### Electrochemical oxygen reduction reaction (ORR)

Electrochemical measurements were performed using a three-electrode system (Pine Research Instrumentation) at 30 °C, with a rotating disk electrode (RDE, 0.196 cm^2^) connected to an Autolab PGSTAT302N electrochemical workstation. A Pt foil (1 × 1 cm^2^) and a Ag/AgCl (3 M) electrode (calibrated before use) were used as the counter and reference electrodes, respectively. All potentials were converted into values with reference to a reversible hydrogen electrode (RHE). The ultrathin Pt nanostructures were supported on carbon black (Ketjen Black EC-300J) with a metal loading of ∼20% (precise loading determined by ICP-MS). Commercial Pt/C (JM, 20% Pt/XC72R, HiSPEC 3000) was used as a reference catalyst. The catalysts were dispersed in a mixture of water, isopropanol and 5% Nafion (volume ratio, 4 : 1 : 0.02) under ultrasonication for 1 h, producing a homogeneous ink with a Pt concentration of 0.16 mg mL^–1^. Then, 12.5 μL of the ink (Pt, 2 μg) was dropped and rotationally dried onto a pre-cleaned glassy carbon RDE.^63^ The catalyst on the electrode was activated before the electrochemical measurement by CV scanning between 0 and 1.2 V for 20 cycles at a rate of 500 mV s^–1^ in N_2_-saturated HClO_4_ (0.1 M). The CV curves were recorded in N_2_-saturated HClO_4_ (0.1 M) in the potential range of 0.05–1.2 V at a scanning rate of 50 mV s^–1^. The ECSAs were calculated based on the charges associated with the adsorption of monolayer hydrogen on the Pt surface in the region of 0.05–0.4 V after double-layer correction with a reference value of 210 C cm^–2^. ORR polarization curves of the catalysts were measured in the potential range of 0.05–1.03 V in O_2_-saturated HClO_4_ (0.1 M) at a scanning rate of 10 mV s^–1^ and a rotating speed of 1600 rpm. *iR* compensation and background current correction were applied to the ORR measurements. The accelerated durability tests were performed by 10 000 cycles of potential sweeps (0.6–1.1 V, 0.1 V s^–1^) in O_2_-saturated HClO_4_ (0.1 M). The CVs and ORR polarization curves were measured after the accelerated durability tests.

### Characterizations

Transmission electron microscopy (TEM) was performed with a Hitachi HT-7700 microscope equipped with a LaB_6_ filament, operated at 100 kV. High-resolution TEM (HRTEM) and high-angle annular dark field scanning transmission electron microscopy (HAADF-STEM) were performed on an FEI Tecnai F20 FEG-TEM microscope operated at 200 kV. X-ray diffraction (XRD) patterns were recorded on a Rigaku SmartLab Powder X-ray diffractometer equipped with Cu Kα radiation and D/teX Ultra detector, scanning from 30 to 90° (2θ) at a rate of 5° min^–1^. Inductively coupled plasma mass spectrometry (ICP-MS) was performed on an Agilent 7500CE.

## Conflicts of interest

There are no conflicts to declare.

## Supplementary Material

Supplementary information
