# Supplementary material for: Synthesis of ultrathin platinum nanoplates for enhanced oxygen reduction activity
Source: Chem Sci. 2017 Oct 30;9(2):398–404. doi: 10.1039/c7sc02997g (PMC5868310; doi:10.1039/c7sc02997g)
Supplement: Supplementary file 1 [file SC-009-C7SC02997G-s001.pdf]

Electronic Supplementary Material (ESI) for Chemical Science.
This journal is © The Royal Society of Chemistry 2017

Supplementary Information (ESI)

Synthesis of Ultrathin Platinum Nanoplates for Enhanced Oxygen Reduction Activity

Hongpo Liu,[a] Ping Zhong,[a] Kai Liu,[a] Lu Han,[b] Haoquan Zheng,[c] Yadong Yin,*[d] and Chuanbo Gao*[a]

[a] Center for Materials Chemistry, Frontier Institute of Science and Technology, and State Key Laboratory of Multiphase Flow in Power Engineering, Xi'an Jiaotong University, Xi'an, Shaanxi 710054, China; [b] School of Chemistry and Chemical Engineering, Shanghai Jiao Tong University, Shanghai 200240, China; [c] School of Chemistry and Chemical Engineering, Shaanxi Normal University, Xi'an, Shaanxi 710119, China; [d] Department of Chemistry, University of California, Riverside, California 92521, United States.
Correspondence: gaochuanbo@mail.xjtu.edu.cn (C.G.), yadong.yin@ucr.edu (Y.Y.)

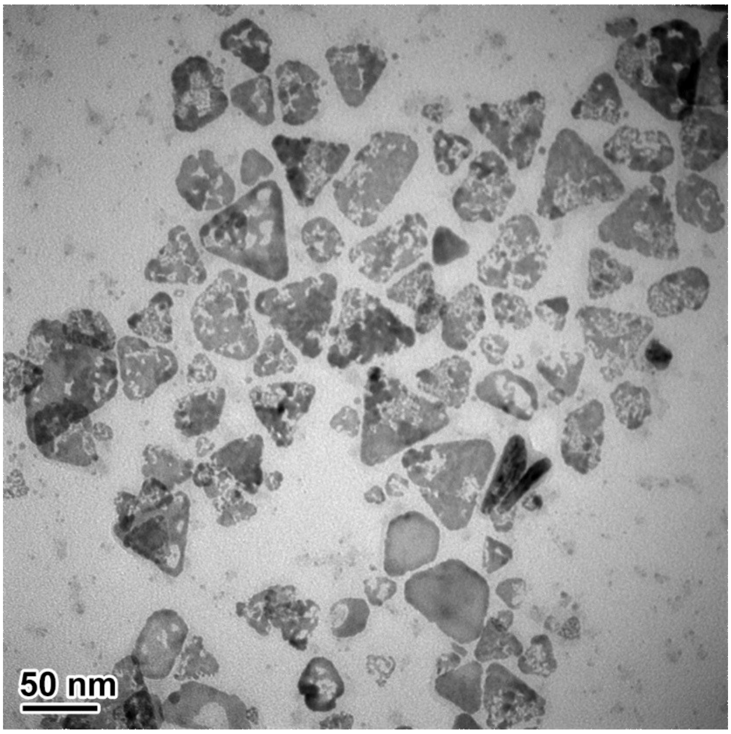

Figure S1. TEM image of the hollow Ag/Pt nanoplates obtained by galvanic replacement between Ag nanoplates and H_2PtCl_6.

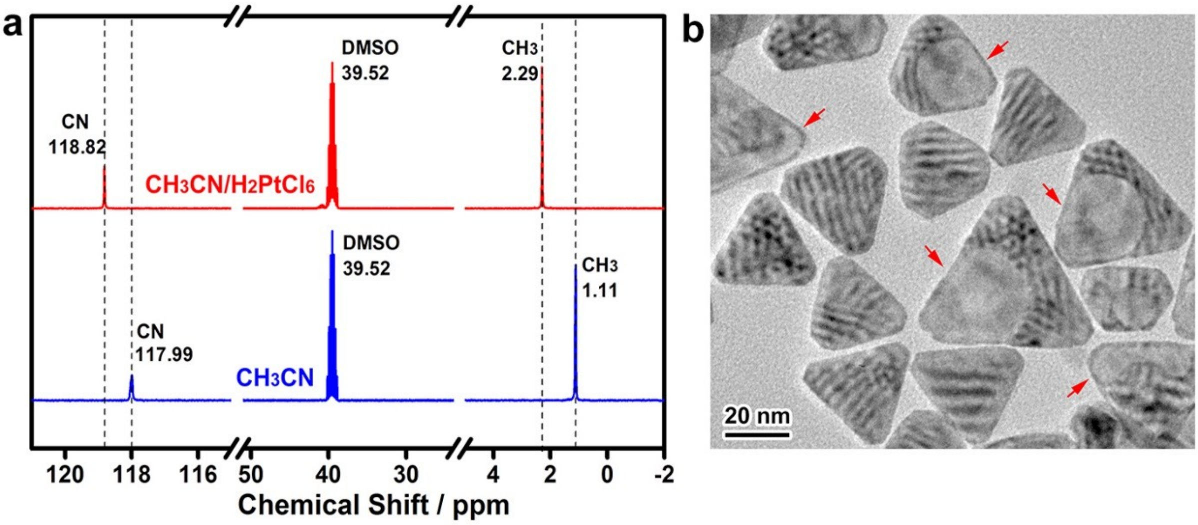

Figure S2. (a) ^{13}C NMR spectra of CH_3CN before and after coordinating to H_2PtCl_6. (b) TEM images of the Ag@Pt core/shell nanoplates synthesized in the absence of CH_3CN. Arrows indicate voids formed by the galvanic replacement.

Discussion on Figure S2: Acetonitrile was chosen as the ligand, which proved to be effective in coordinating to the Pt(IV) salt, as revealed by ^{13}C NMR spectroscopy (Figure S2a). The ^{13}C in –CN and –CH_3 of acetonitrile after coordination produce signals at 118.82 and 2.29 ppm of the chemical shifts, respectively, which are on the low-field side of the corresponding signals from pure acetonitrile, *i.e.* 117.99 and 1.11 ppm, respectively. It indicates significant electron transfer from acetonitrile to Pt, consistent with the formation of the coordination bonding. The effect of the ligand on the galvanic replacement can be confirmed experimentally. As revealed by TEM (Figure S2b), the epitaxial growth of Pt on Ag nanoplates in the absence of acetonitrile leads to significant etching of the Ag nanoplates, which is a clear sign of the galvanic replacement.

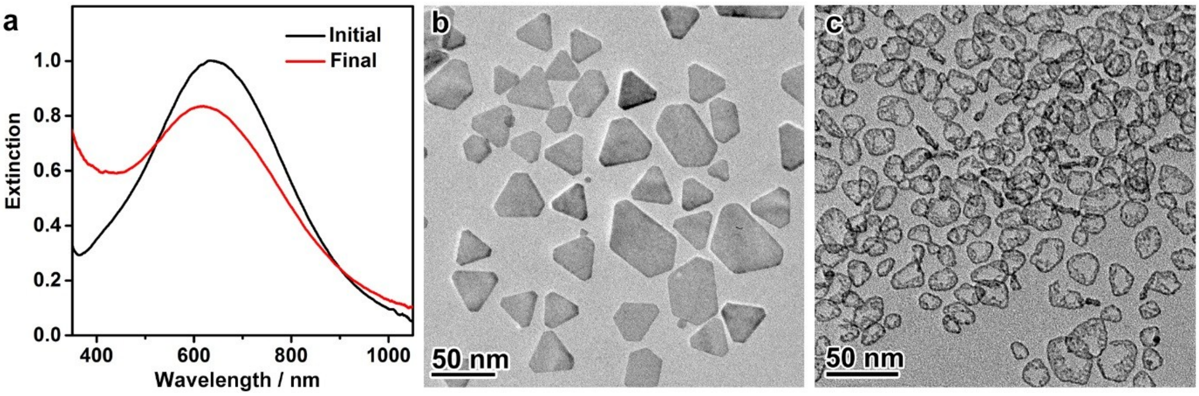

Figure S3. Synthesis of Ag@Pt core/shell nanoplates and ultrathin Pt nanoplates following a typical synthesis except for additional sulfite (SO_3^{2-}) as a ligand to the chloroplatinate salt. (a) UV-vis spectra of the Ag nanoplates before and after epitaxial growth of Pt. (b) TEM image of the resulting Ag@Pt core/shell nanoplates. (c) TEM image of the Pt nanostructure after removing the Ag template.

Discussion on Figure S3: It can be inferred that although deprotonated ascorbic acid and high-temperature H_2 have been employed as strong reducing agents, the reduction of the Pt(IV)/SO_3^{2-}

complex is still very difficult to induce continuous growth of Pt on the Ag nanoplates. As a result, only an unfavorably thin layer of Pt has been grown on the Ag nanoplates, which can be evidenced by the retention of the LSPR of the Ag nanoplates after the Pt deposition (Figure S3a). Instead of obtaining ultrathin Pt nanoplates with smooth surface and well-defined crystal facets, only nanoframes can be obtained from this synthesis (Figure S3c). Therefore, SO_3^{2-} represents a ligand that forms a stable complex with the chloroplatinate salt, favorable for the suppression of the galvanic replacement. However, the complex turned out to be too stable to be readily reduced by common reducing agents, and thus is inappropriate for templated synthesis of ultrathin Pt nanostructures. A balance between the coordination effect of the ligand and the reducibility of the resulting complex should be taken into consideration for a successful synthesis.

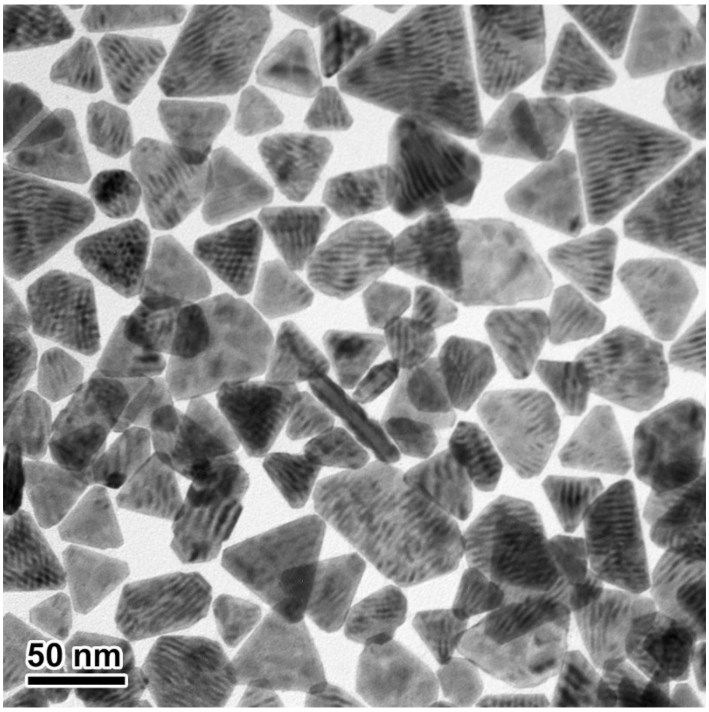

Figure S4. TEM image of the Ag@Pt core/shell nanoplates synthesized from a typical synthesis, except for the use of NO_2^- ($NaNO_2$) as the ligand in place of CH_3CN.

Discussion on Figure S4: It can be inferred that both NO_2^- and CH_3CN leads to continuous epitaxial growth of an ultrathin layer of Pt on Ag nanoplates to form Ag@Pt core/shell nanoplates, with Moiré fringes clearly observed in both cases. Both ligands can form complexes with the chloroplatinate salt to reduce the reduction potential of Pt salt so that the tendency of the galvanic replacement has been suppressed, and the complexes are both not too stable for to allow their convenient reduction and thus continuous epitaxial growth. This result confirms the importance of the balancing the coordination effect of the ligand and the reducibility of the resulting complex for a successful synthesis, and provides an alternative ligand that works in the templated synthesis of ultrathin Pt nanostructures.

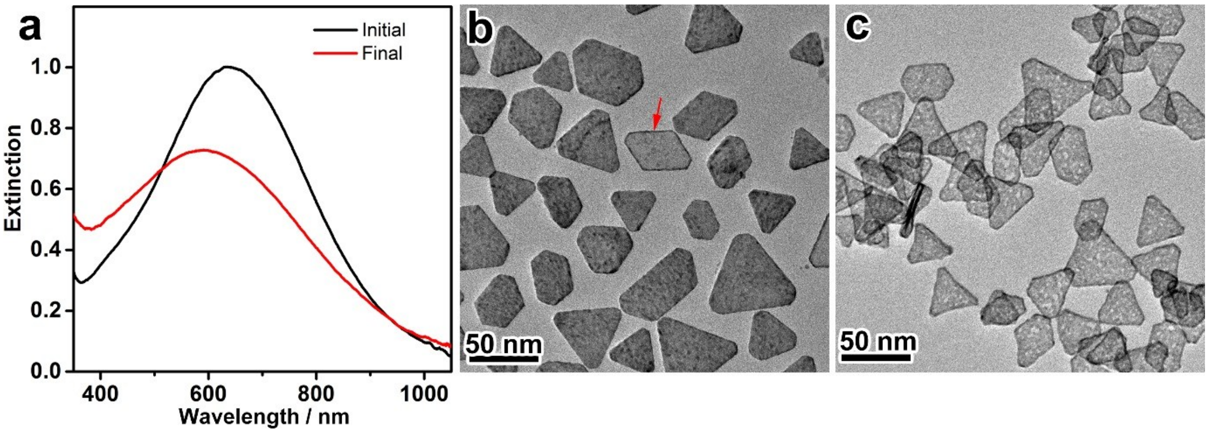

Figure S5. Synthesis of Ag@Pt core/shell nanoplates and ultrathin Pt nanoplates with deprotonated ascorbic acid as the reducing agent, in the absence of high-temperature H_2. (a) UV-vis spectra of Ag nanoplates before and after the growth of Pt for 12 h at 120 °C. (b) TEM image of the Ag@Pt core/shell nanoplates synthesized in the absence of CH_3CN. The arrow indicates a void formed by the galvanic replacement. (c) TEM image of the Pt nanostructure obtained after etching of the Ag template.

Discussion on Figure S5: After the epitaxial growth in this control experiment, the LSPR of the Ag nanoplates underwent a blue shift in the band position and a decrease in the extinction efficiency (Figure S5a). However, the resulting core/shell nanostructure still showed remarkable LSPR property of Ag, suggesting that only a very thin layer of Pt has been grown on the Ag nanoplates. Instead of forming ultrathin Pt nanoplates with smooth surface and well-defined facet, this synthesis affords ultrathin Pt nanoplates with high porosity and deformed morphology (Figure S5c). It can be inferred that in the absence of high-temperature H_2, Pt cannot continuously grow on the Ag nanoplates in a favorable kinetics, leading to Pt nanostructures with an unfavorably thin thickness and thus poor stability in practical applications.

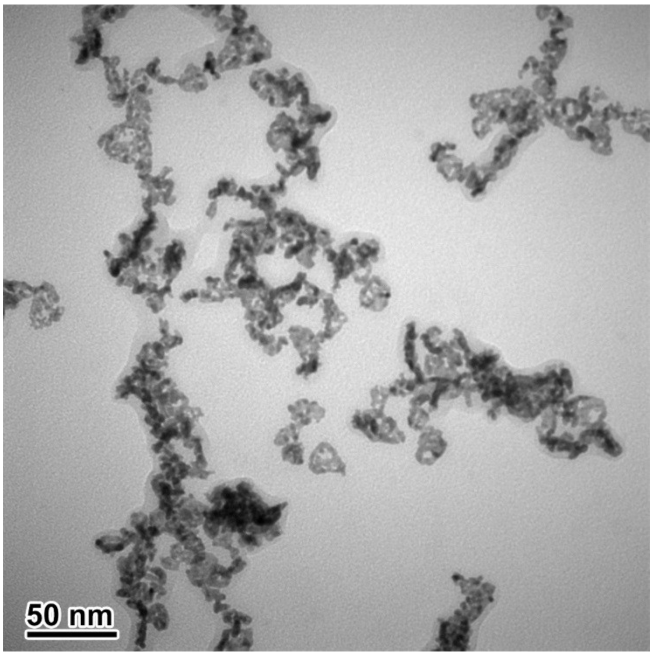

Figure S6. TEM image of the product obtained from a typical synthesis in the absence of ascorbic acid, with only high-temperature H_2 as the reducing agent.

Discussion on Figure S6: Only hollow Ag/Pt nanoplates have been obtained from this synthesis. Although high-temperature H_2 represent a strong reducing agent, its reduction reaction with the Pt(IV) salt occurs at the air/water interface, which makes it a significantly sluggish process. As acetonitrile by itself could not completely prevent the galvanic replacement, at the first stage of the epitaxial growth, galvanic replacement readily occurred to produce hollow nanostructures, due to the significantly sluggish kinetics of chemical reduction and thus crystal growth compared with the galvanic replacement. It therefore confirmed that protonated ascorbic acid is still necessary for the templated synthesis of ultrathin Pt nanoplates despite of the use of high-temperature H_2 as the reducing agent.

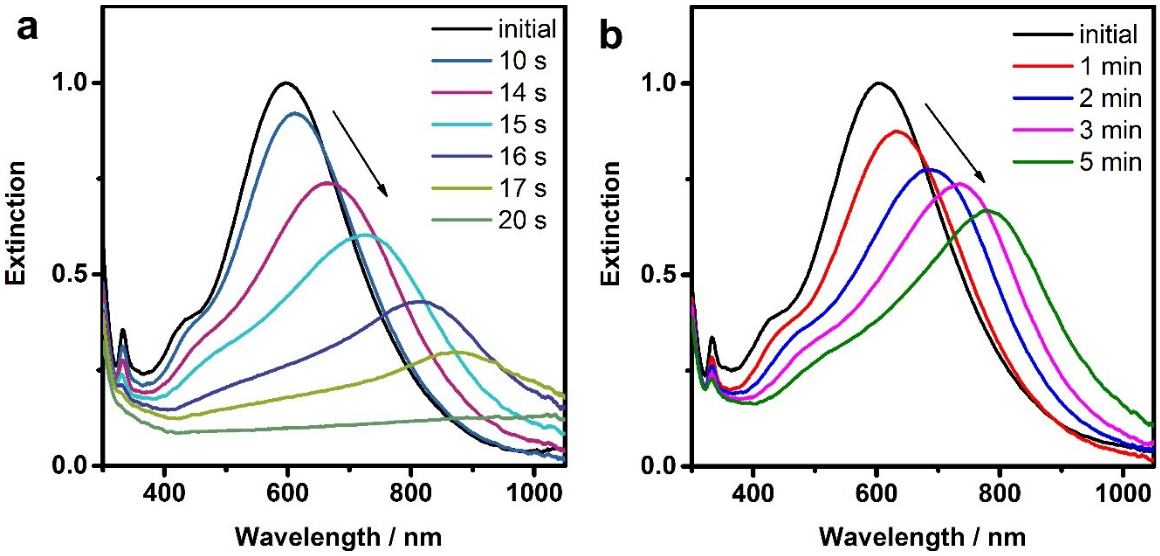

Figure S7. Evolution of the UV-vis spectroscopy of the Ag nanoplates upon mixing with H_2PtCl_6, (a) in the absence of PVP and (b) in the presence of PVP.

Discussion on Figure S7: It is inferred that polyvinylpyrrolidone (PVP) can adsorb on the Ag surface, and therefore suppressed the galvanic replacement from the perspective of the reaction kinetics. Therefore, in all our experiments, PVP has been introduced as a capping agent to enhance the stability of the Ag nanoplates.

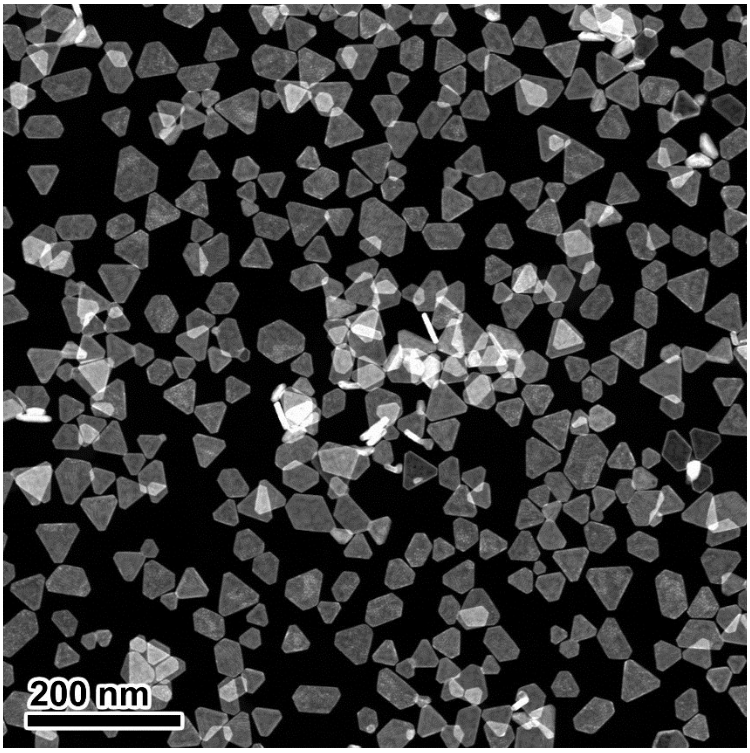

Figure S8. A low-magnification HAADF-STEM image of the Ag@Pt core/shell nanoplates.

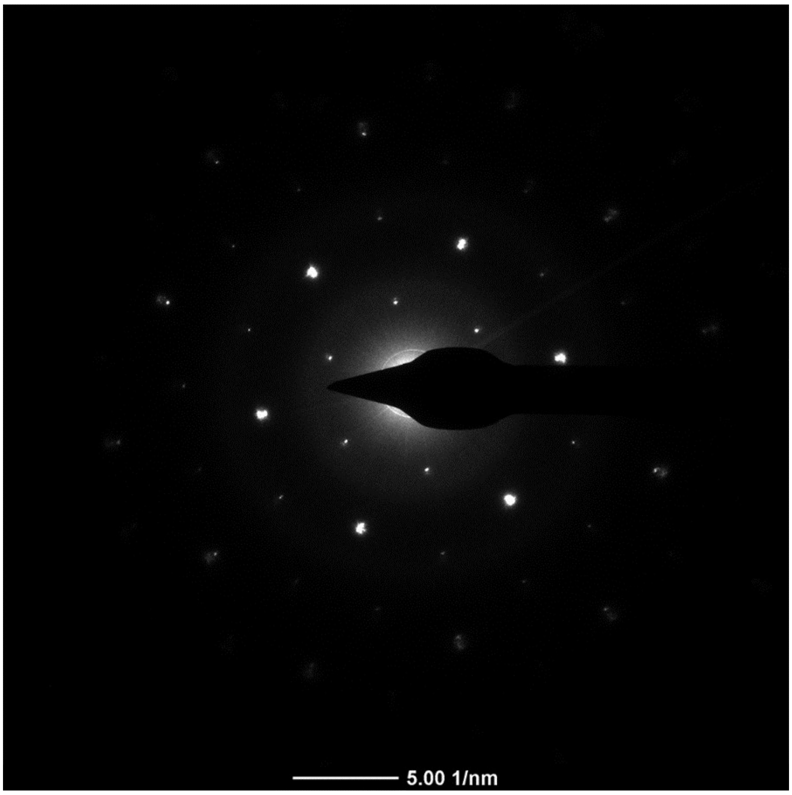

Figure S9. Electron diffraction pattern of a Ag@Pt core/shell nanoplate. A part of this image has been shown in Figure 2b.

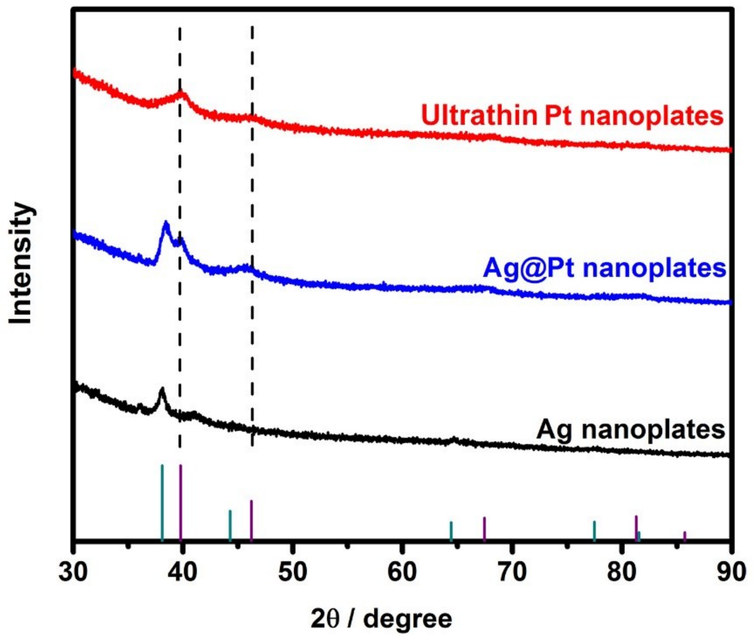

Figure S10. XRD patterns of the Ag nanoplates, Ag@Pt core/shell nanoplates and ultrathin Pt nanoplates. Due to the relatively thick thickness of the Pt layer on the Ag nanoplates, reflections from the Pt layer can be clearly detected. After etching the Ag templates, ultrathin Pt nanoplates display intrinsic reflections from Pt.

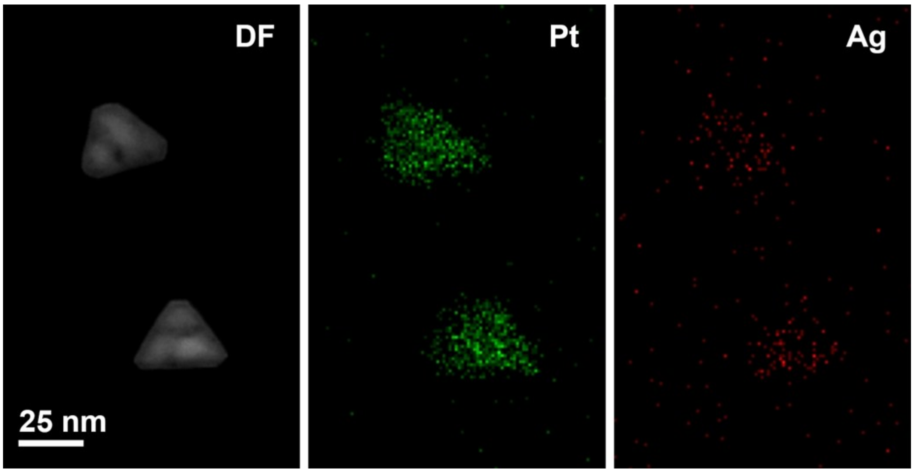

Figure S11. EDS elemental mapping of the ultrathin Pt nanoplates.

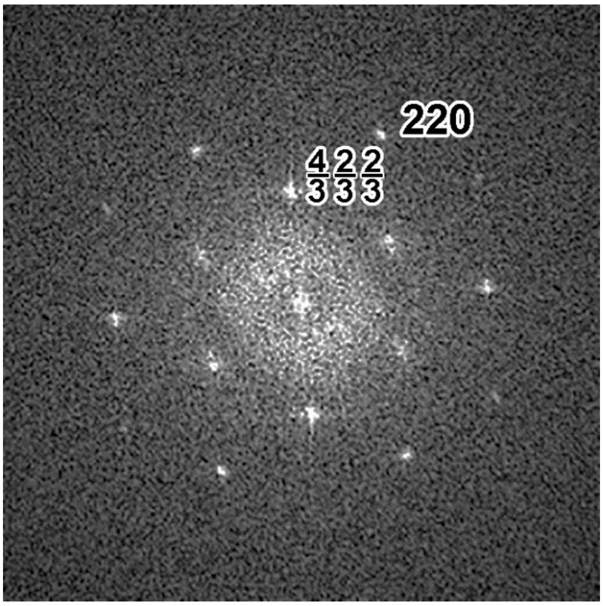

Figure S12. Fourier transform of the HRTEM image of the ultrathin Pt nanoplates (Figure 3c). This pattern is consistent with the electron diffraction pattern (Figure 3d), confirming that the ultrathin Pt nanoplates expose the {111} facet.

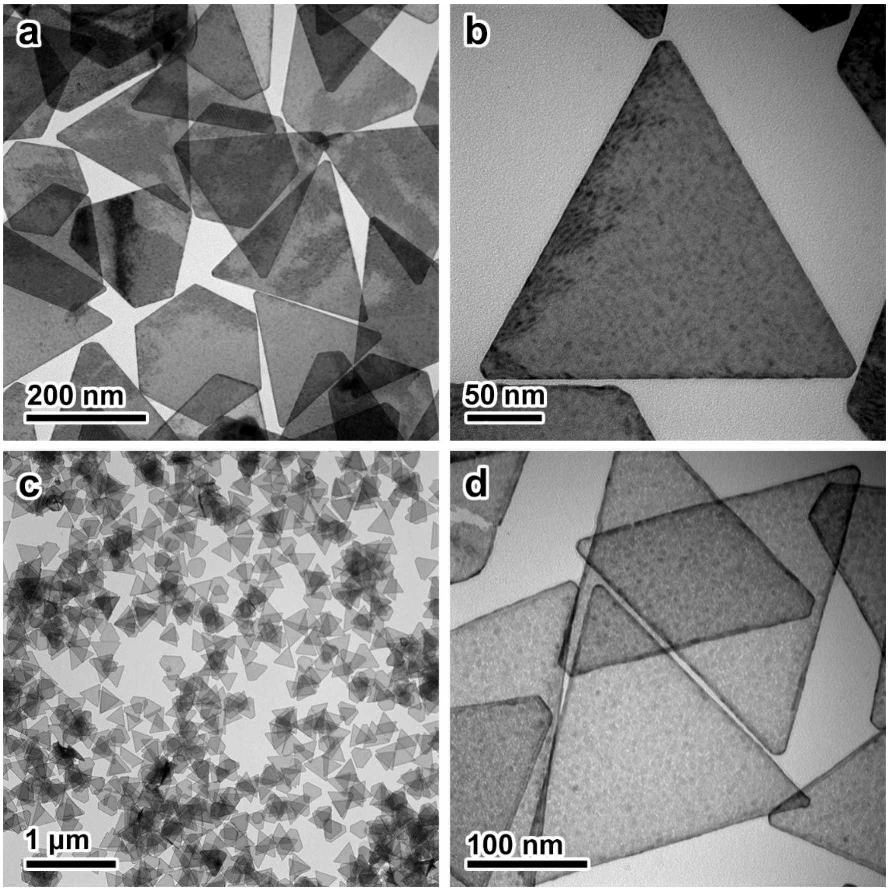

Figure S13. Synthesis of ultrathin Pt nanoplates of a large size (~ 240 nm). Reduction conditions: deprotonated ascorbic acid + H_2 (120 °C). (a–b) TEM images of the Ag@Pt core/shell nanoplates. (c–d) HRTEM images of the ultrathin Pt nanoplates after removal of the

Ag templates. It confirms the versatility of the hard-templating strategy in affording ultrathin Pt nanostructures with different morphologies and sizes.

Synthesis procedures: Ag nanoplates of a large size (~ 240 nm) were synthesized according to our previous report.[1] Typically, Ag nanoplates were centrifuged from 80 mL of the stock solution (see Experimental section), washed with H_2O, and redispersed in 8 mL of H_2O for use as the seeds. TSC (0.075 M, 1 mL), ascorbic acid (0.5 M, 0.3 mL) and CH_3CN (50 mL) were dissolved in 100 mL of H_2O at 5 °C. To this solution were quickly injected $AgNO_3$ (0.1 M, 1.2 mL) and the seed solution under vigorous stirring. After stirring for 30 min, the Ag nanoplates were collected for growth of Pt.

In a typical synthesis of large-size Ag@Pt core/shell nanoplates, 4 mL of PVP (5 wt%), 800 μL of AA (0.5 M), 800 μL of NaOH (1 M) and 200 μL of H_2PtCl_6 (0.1M) were added into 45 mL of the large-size Ag nanoplates (~ 240 nm) in sequence. The reaction system was transfer to a high-pressure tube, filled with H_2 of atmospheric pressure, and stirred at 120 °C for 12 h. Finally, Ag@Pt core/shell nanoplates (~240 nm) were collected by centrifugation, washed twice with H_2O, and redispersed in 10 mL of H_2O.

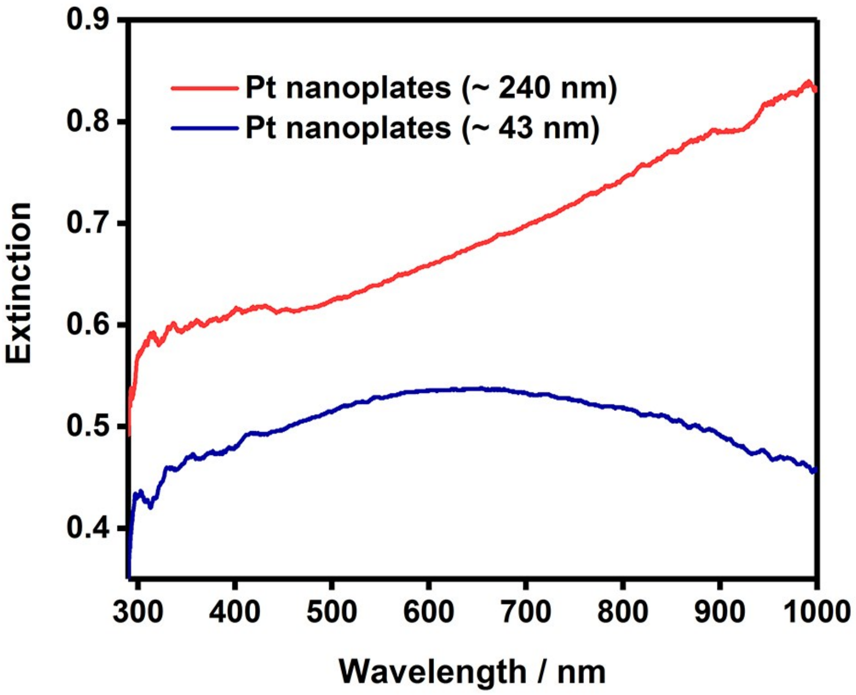

Figure S14. UV-vis spectra of the ultrathin Pt nanoplates of different sizes. Due to the high anisotropy of the ultrathin Pt nanoplates, they showed weak localized surface plasmon resonance property in the visible and near-infrared range of the spectrum. A vague bluish color can be distinguished from their solutions.

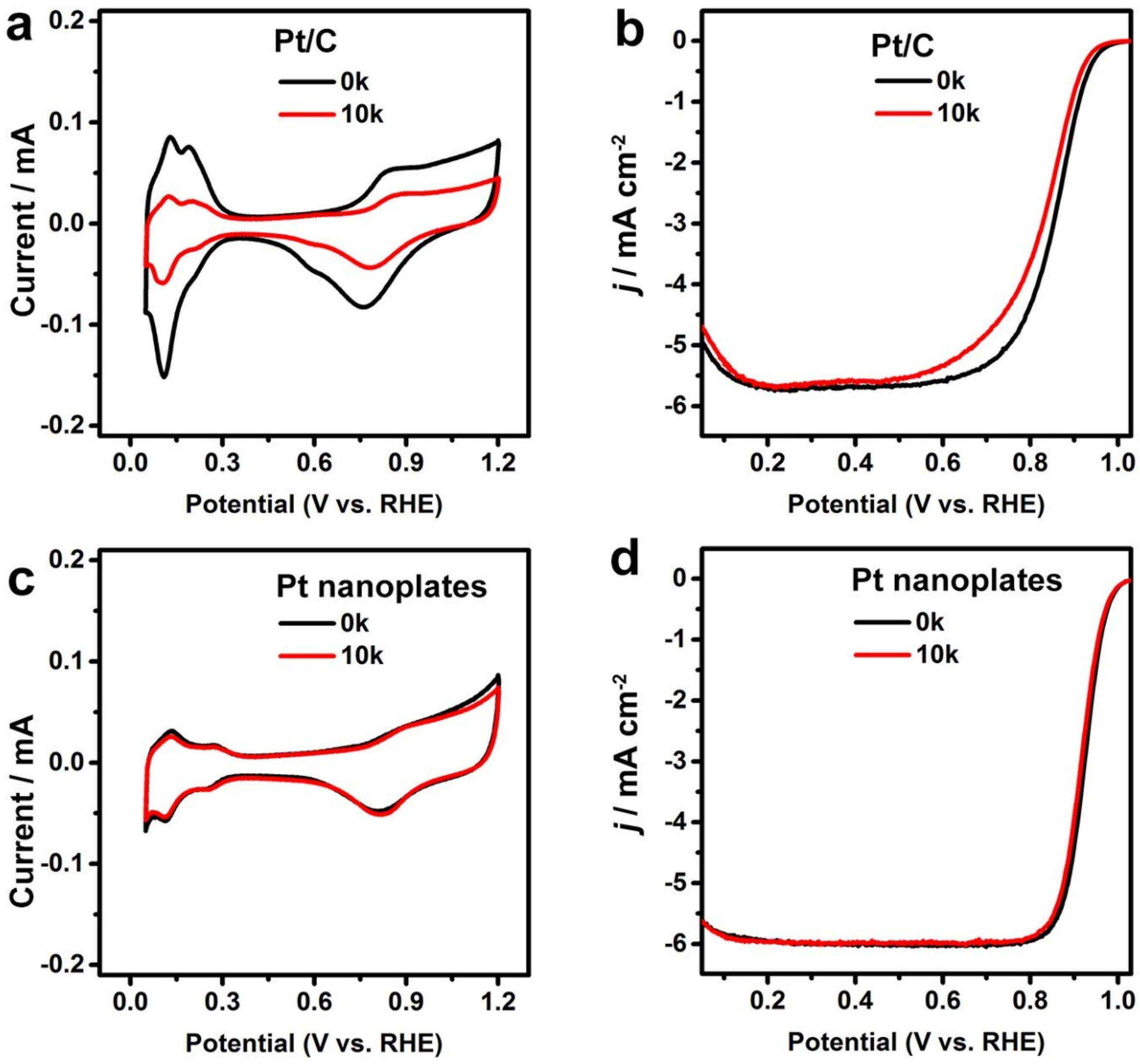

Figure S15. Stability of ultrathin Pt nanoplates in electrocatalytic ORR, in comparison with the commercial Pt/C catalyst. (a, c) CV curves of the catalysts recorded in N_2-saturated $HClO_4$ (0.1 M) at a sweep rate of 50 mV·s^{-1}, before and after 10,000 cycles of the potential sweep in O_2-saturated $HClO_4$ (0.1 M) (0.6–1.1 V, 0.1 V·s^{-1}). (b, d) ORR polarization curves of the catalysts recorded in O_2-saturated $HClO_4$ (0.1 M) at a sweep rate of 10 mV·s^{-1} and a rotation rate of 1600 rpm, before and after 10,000 cycles of the potential sweep in O_2-saturated $HClO_4$ (0.1 M) (0.6–1.1 V, 0.1 V·s^{-1}). The current densities were normalized to the geometric area of the rotating disk electrode (0.196 cm^2).

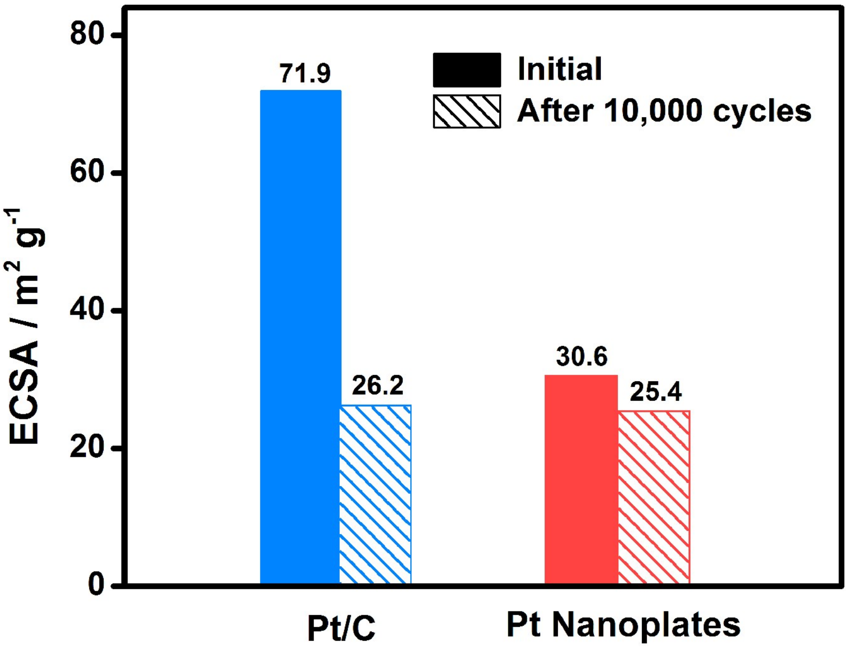

Figure S16. Change of the ECSA of ultrathin Pt nanoplates and the commercial Pt/C catalyst in ORR. These data were summarized based on Figure S15 (a, c).

Discussion on Figure S15, S16: From Figure S15 (a, c) and Figure S16, it can be concluded the ultrathin Pt nanoplates have retained the most of their ECSAs (~83.0%) during the cycling process due to their self-supported nanostructure. For comparison, a significant decrease in the ECSA of the commercial Pt/C has been observed (retaining ~36.4%). Therefore, the ultrathin Pt nanoplates are superior in retaining their ECSA, which promises high catalytic stability.

From Figure S15 (b, d), it can be inferred that a dramatic degradation in the half-wave potential of the ORR curve has been observed with commercial Pt/C after the cycling process, suggesting a poor electrocatalytic stability. In clear contrast, only very minor degradation has been observed in the half-wave potential of the ORR curves with catalysts of the ultrathin Pt nanoplates, thus confirming their remarkable catalytic stability.

Reference:

[1] Liu, X., Li, L., Yang, Y., Yin, Y. & Gao, C. One-step growth of triangular silver nanoplates with predictable sizes on a large scale. *Nanoscale,* **2014,** *6,* 4513-4516.
